# Repair of Postoperative Cervical Pseudomeningocele with the Use of Bone Morphogenetic Protein

**DOI:** 10.7759/cureus.5200

**Published:** 2019-07-22

**Authors:** Dejan Slavnic, Robert Mccabe, Doris Tong, Teck M Soo

**Affiliations:** 1 Neurosurgery, Ascension Providence Hospital, Michigan State University, College of Human Medicine, Southfield, USA

**Keywords:** cervical spine, csf leak, pseudomeningocele, bone morphogenetic protein, bmp

## Abstract

Recurrent cerebrospinal fluid (CSF) leak carries significant morbidity and mortality. A 25-year-old Caucasian male developed a symptomatic pseudomeningocele eight days after surgical resection of a cervical schwannoma. With no improvement after lumbar drain placement, the dural sleeve defect over the left C3 nerve root was repaired with suturable DuraGen® (Integra LifeSciences, Plainsboro, NJ, USA). Muscle and fat graft were then laid over the repair site and covered by Tisseel® (Baxter Healthcare, Deerfield, IL, USA). Recombinant human bone morphogenetic protein-2 (rhBMP-2) was applied via extra-small sponge laid over the graft. At 15-month clinical and 16-month radiographic follow-up, the patient had complete resolution of symptoms without any evidence of infection, ectopic bone formation, excessive inflammation, neoplasm, or recurrent CSF leak. This case demonstrates the successful use of BMP in the treatment of recurrent symptomatic cervical pseudomeningocele after tumor resection. We believe that the pro-inflammatory effects of rhBMP-2 lead to early scarring of dural defect and resolution of CSF leak.

## Introduction

Cerebrospinal fluid (CSF) leak after spinal surgery may lead to significant morbidity including CSF fistulas, meningitis, brain abscess, intracranial hemorrhage, and neurologic deficits [1,2]. The incidence of CSF leak varies depending on the procedure performed, but it is generally higher with open spine approaches [3]. In instances where the dura is violated without injury to the arachnoid membrane, a true meningocele lined with the arachnoid membrane may develop. However, in dural-arachnoid tears, such as during intradural tumor resection, extravasation of CSF outside of the thecal sac will inadvertently form a pseudomeningocele, a CSF pseudocyst lined by fibrous tissue [4,5]. 

We present a case of recurrent CSF leak and a symptomatic pseudomeningocele formation after resection of a cervical schwannoma in a 25-year-old male who later underwent surgical repair with the use of bone morphogenetic protein (BMP). BMPs have been used successfully to treat recurrent CSF leak after transsphenoidal surgery [6]. In our institution, we have effectively applied recombinant human bone morphogenetic protein-2 (rhBMP-2) for the repair of persistent CSF leak secondary to encephalocele. BMPs are signaling molecules that belong to a superfamily of proteins known as transforming growth factor-β (TGF-β) that are mostly involved in osteogenesis [7,8]. When used in high quantities BMPs were associated with complications such as ectopic bone growth, osteolysis, and systemic neoplasm [9]. We sought to demonstrate that BMPs could be used in the repair of recurrent CSF leak and pseudomeningocele without long-term complications.

## Case presentation

**Figure 1:**
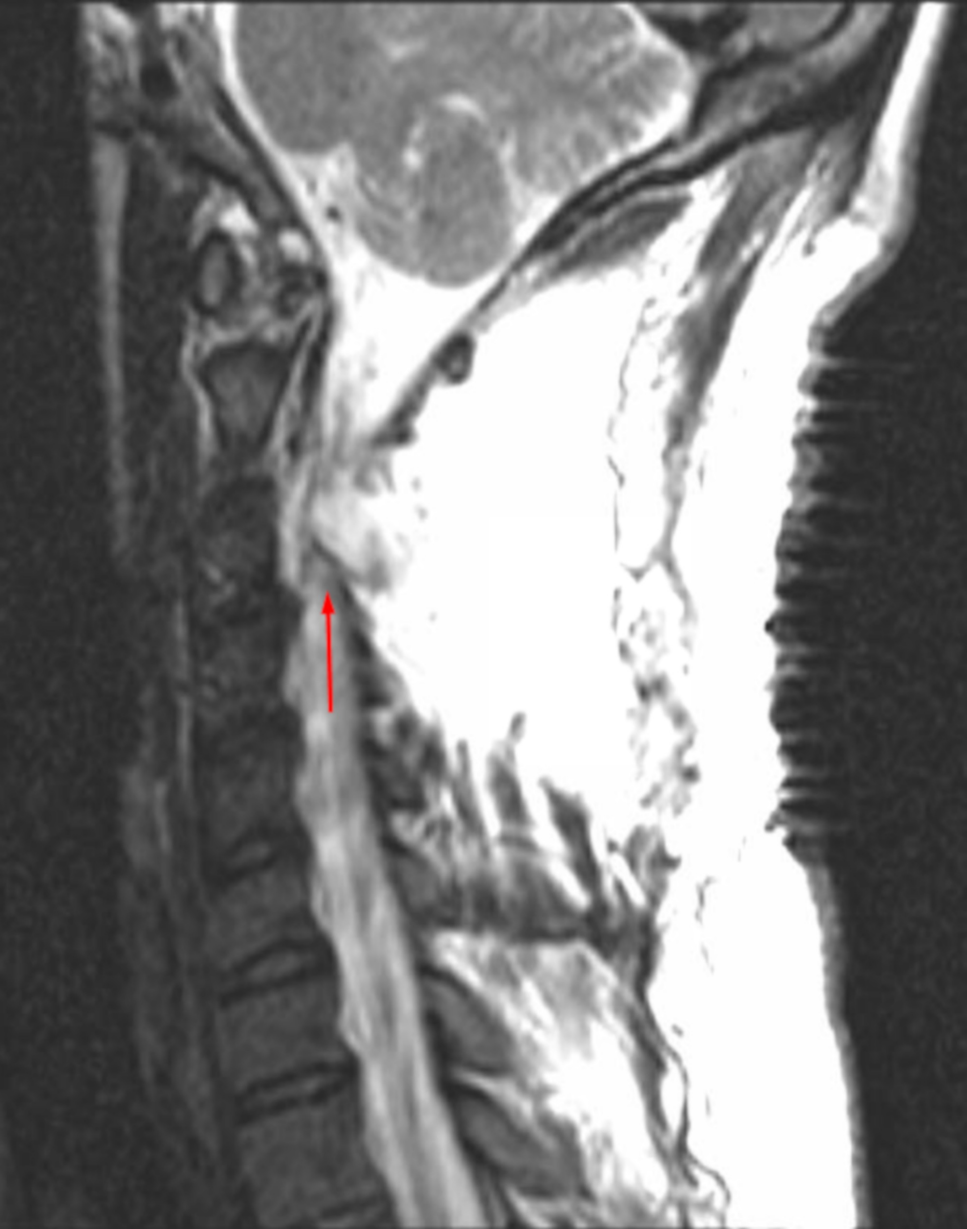
Cervical spine sagittal MRI without contrast demonstrating a large pseudomeningocele within the surgical site prior to dural repair with BMP MRI (Magnetic Resonance Imaging), BMP (Bone Morphogenetic Protein)

A 25-year-old male without significant past medical history presented to the neurosurgery clinic with worsening left shoulder and neck pain. Magnetic resonance imaging (MRI) revealed a 2.6 x 1.1 cm intradural extramedullary mass within the left C2-3 neural foramen consistent with schwannoma. The patient underwent left C2-3 hemilaminectomy with complete resection of the tumor. The 2 x 1 cm dural defect was repaired in a water-tight fashion with suturable collagen matrix (DuraGen®, Integra LifeSciences, Plainsboro, NJ, USA) and covered by fibrin sealant (Tisseel®, Baxter Healthcare, Deerfield, IL, USA). There were no complications, and he was sent home four days later. On postoperative day eight, the patient returned to the emergency room with symptoms of nausea, vomiting, headache, and photophobia that worsened when he was upright. MRI showed a large pseudomeningocele at the surgical site (Figure 1) for which lumbar drain was placed. The patient’s symptoms returned after the drain was removed five days later. Therefore, the decision was made to proceed with surgical exploration and repair. 

A lumbar drain was inserted at the beginning of surgery. The posterior cervical incision was opened and explored. The 2 x 1 cm dural defect was localized, and suturable DuraGen® was used to repair the defect completely. A piece of fascia and fat graft were laid over the dura and covered by Tisseel®. The 2 x 2 cm absorbable sponge from the extra-small BMP kit was soaked with 1.4 cc rhBMP-2 and laid over the top. The suction drain (Hemovac®, Zimmer Biomet, Warsaw, IN, USA) was inserted, and the wound closed in a standard anatomical layer fashion without any complications.

**Figure 2:**
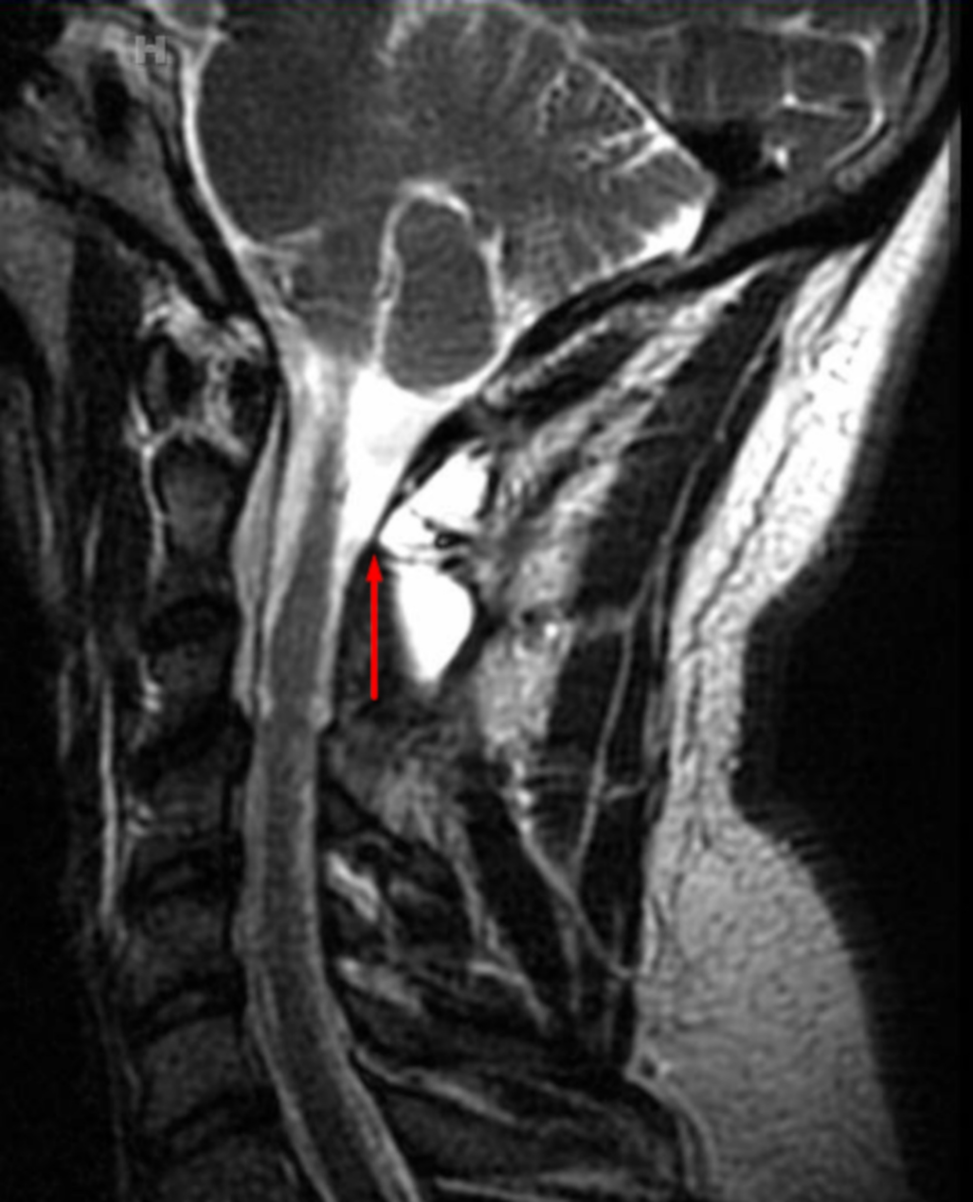
Cervical spine sagittal MRI without contrast showing a near-complete resolution of the pseudomeningocele after dural repair with BMP MRI (Magnetic Resonance Imaging), BMP (Bone Morphogenetic Protein)

The lumbar drain remained in place and was open to drain for five days. The patient was discharged home on postoperative day six. He was followed clinically for 15 months after surgery. The MRI at 16 months post-surgery showed no evidence of recurrent tumor, CSF leak, infection, extraneous bony growth, excessive inflammation, or neoplasm (Figure 2).

## Discussion

Spinal intradural tumor resection surgeries carry 5-18% chance of CSF-related complications, including the development of CSF fistula, pseudomeningocele, and meningitis. The complications and their respective treatments of prolonged bed rest, re-operation, and extended use of antibiotics carry significant morbidity and mortality [10]. One of the most widely used treatments for spinal CSF leak is direct suture repair of the dural tear, with a still significant failure rate of 5-9% [1]. In patients with surgically inaccessible or irreparable dural tears, a CSF sealant such as Tisseel® or DuraSeal® may be applied, with a failure rate of 5% [11]. Another well-known treatment is the placement of the autologous fat graft over the CSF leak defect which eliminates the dead space created by the laminectomy and muscle dissection [10,12]. 

Here we present a case of successful symptomatic CSF leak and pseudomeningocele repair with the use of BMP in a patient who underwent cervical schwannoma resection. BMPs belong to a TGF-β superfamily of signaling molecules discovered in 1965 [7]. Their involvement is most significant in the early embryological development of mammals as well as regulation of osteogenesis [8]. In 2002, the rhBMP-2 was approved by the United States Food and Drug Administration (US FDA) for use in lumbar fusions due to its osteoinductive properties [13]. An article by Huang et al. demonstrates that rhBMP-2 induces an inflammatory state that was evident on histologic tissue sections and systemic blood samples of rats with subcutaneously implanted BMP [14]. It is our extrapolation that this pro-inflammatory BMP-induced response leads to quicker scarring and healing of the CSF leak defect preventing the reoccurrence of symptomatic pseudomeningocele formation.

## Conclusions

In our study, none of the reported complications associated with BMP use were observed. Although BMPs are scrupulously investigated signaling molecules, more research is required to fully understand their function and use in surgery, wound healing, and tissue repair. Future studies should focus on further demonstrating the safety of BMP in the treatment of CSF leak through stronger study designs, such as a randomized controlled trial.
